# Trends and inequalities in antenatal care coverage in Benin (2006–2017): an application of World Health Organization’s Health Equity Assessment Toolkit

**DOI:** 10.1186/s12913-024-11261-z

**Published:** 2024-09-04

**Authors:** Richard Gyan Aboagye, Joshua Okyere, Josephine Akua Ackah, Edward Kwabena Ameyaw, Abdul-Aziz Seidu, Bright Opoku Ahinkorah

**Affiliations:** 1 School of Population Health, University of New South Wales, Sydney, Australia; 2 Department of Family and Community Health, Fred N. Binka School of Public Health, University of Health and Allied Sciences, Hohoe, Ghana; 3 Department of Population and Health, University of Cape Coast, Cape Coast, Ghana; 4 Department of Population Health, London School of Hygiene and Tropical Medicine, London, UK; 5 Institute of Policy Studies and School of Graduate Studies, Lingnan University, Lingnan, Hong Kong China; 6 L & E Research Consult Ltd, Wa, Upper West Region Ghana; 7 College of Public Health, Medical and Veterinary Sciences, James Cook University, Townsville, QLD 4811 Australia; 8 REMS Consultancy Services Limited, Sekondi-Takoradi, Western region Ghana; 9 School of Public Health, Faculty of Health, University of Technology Sydney, Sydney, Australia; 10 School of Clinical Medicine, University of New South Wales Sydney, Sydney, Australia

**Keywords:** Antenatal care, Benin, Demographic and Health Survey, Inequalities, Trends

## Abstract

**Introduction:**

Between 2006 and 2017, antenatal care (ANC) coverage in Benin declined, potentially exacerbating inequalities and substantiating the need for health inequality monitoring. This study examines inequalities in ANC attendance in Benin, disaggregated by women’s age, educational level, economic status, place of residence, region of residence, and the extent to which they have changed over time.

**Methods:**

Three rounds of the Benin Demographic and Health Surveys (2006, 2011–12, and 2017–18) were analyzed to examine inequalities in ANC coverage. An exploratory descriptive approach was adopted for the analysis. Simple [difference (D) and ratio (R)] and complex [population attributable risk (PAR) and population attributable fraction (PAF)] measures of inequalities were computed using the World Health Organization’s Health Equity Assessment Toolkit (WHO’s HEAT) online platform. The measures were computed separately for each of the three surveys, and their estimates were compared.

**Results:**

The findings revealed an 8.4% decline in at least four ANC visits between 2006 and 2017–18. The decline occurred irrespective of age, educational status, economic status, place of residence, and region. Region-related inequalities were the largest and increased slightly between 2006 (D = 54.6; *R* = 2.6; PAF = 47.8, PAR = 29.0) and 2017–18 (D = 55.8; *R* = 3.1; PAF = 57.2, PAR = 29.8). Education (**2006**: D = 31.3, *R* = 1.6, PAF = 40.5, PAR = 24.5; **2017–18**: D = 25.2, *R* = 1.6, PAF = 34.9, PAR = 18.1) and rural-urban (**2006**: D = 16.8, *R* = 1.3, PAF = 17.8, PAR = 10.8; **2017–18**: D = 11.2, *R* = 1.2, PAF = 13.1, PAR = 6.8) inequalities reduced while economic status inequalities did not improve (**2006**: D = 48, *R* = 2.2, PAF = 44.5, PAR = 26.9; **2017–18**: D = 43.9, *R* = 2.4, PAF = 45.0, PAR = 23.4). Age inequalities were very minimal.

**Conclusion:**

ANC inequalities remain deeply ingrained in Benin. Addressing their varying levels requires comprehensive strategies that encompass both supply—and demand-side interventions, focusing on reaching uneducated women in the poorest households and those residing in rural areas and Atacora.

## Introduction

Over the past decades, several studies have provided evidence to support the importance and positive impact of antenatal care (ANC) on maternal, newborn, and child health outcomes [1–5]. ANC is considered as an important element in the maternal continuum of care [6–8] with several models proposing recommended guidelines. Notable are the standard “Western” model of 12 visits, the 2002 World Health Organization (WHO) Focused Antenatal Care model of four visits, and the latest 2016 WHO ANC model of eight visits to ensure positive pregnancy outcomes [5] and applicability in resource-deprived settings. Efforts following these guidelines and their updates have contributed to the current global estimates of 88% for at least one ANC and 66% for at least four ANC visits [9], but with sharp regional differences. The percentage of women aged 15–49 who attend at least four ANC visits was lowest for Western and Central Africa (53%), South Asia (55%), and Eastern and Southern Africa (54%) [9]. The disparities are further exacerbated by women’s socio-economic and demographic characteristics, where lower coverage of ANC attendance is skewed towards those residing in rural areas or specific administrative regions, poor, not educated, and in specific age groups [10, 11].

Within sub-Saharan Africa, countries like Benin have witnessed a decline in ANC coverage. Though an estimated 90% of women (15–49 years) have had at least one ANC visit in Benin [12], values were far lower for those who met the recommended four visits. The proportion reduced from 61.4% in 2006 [13] to 52% in 2018 [12]. For ANC visits of at least eight, Ekholuenetale and colleagues only found the national coverage to be 8 per 100 women, implying a rather slow progress toward institutionalizing the new guideline [14]. As part of ANC, mothers receive the needed micro supplementation, immunization against tetanus, detection of early signs of complications, and medications for endemic health conditions to reduce the risk of pregnancy complications as well as maternal/child deaths [2, 5, 9, 15, 16]. This is particularly important in Benin, where the rates of maternal/child mortality are already high; maternal, infant, and under-five mortality rates stand at 405 deaths per 100,000 live births, 55 deaths per 1000 live births, and 83.5 per 1000 live births, respectively [17, 18]. Benin is also far from reaching the Sustainable Development Goal (SDG) targets for maternal and under-five survival by 2030. As a result, the decline in Benin’s ANC (at least four visits) coverage raises dire public health concerns, highlighting the need to explore underlying factors and inequalities to guide targeted interventions.

Exploring inequalities in the proportion of women who have had at least four ANC visits presents three opportunities – (1) tracking the impact of existing ANC-related health programs, strategies, interventions, and policies on marginalized groups; (2) identifying who to target in new programs and interventions, and (3) guiding research to provide socio-cultural and contextual explanations to the differences observed within groups. Within the context of this study, we prioritize the concept of “health inequality” to highlight differences in health outcomes (at least four ANC visits) within and between social groups. The differences could be a product of avoidable systematic, unjust, and unfair processes or circumstances [19]. For ANC, observed inequalities have been reported for women in different educational, age, wealth, and residential groups [10, 13, 20–22]. According to the United Nations Children’s Fund [23], in Benin, an estimated 66% of women in urban areas had at least four ANC visits compared to 54% in rural areas. Only 35% of women in the poorest households also made at least four ANC visits compared to 83% among those in the richest households. The proportion of women aged 20–34 years who made at least four ANC visits (60%) was 7% more than those aged less than 20 years (53%), while for educated women (87%), the proportion was 36% more than that of uneducated women (51%). Among the regions of residence, ANC coverage of at least four was lowest in Atacora (27%) and Alibori (36%) compared to Littoral (86%). The observed decelerating trends in ANC visits among women in Benin imply that inherent patterns of low uptake will be concentrated in some social groups compared to others. The magnitude of the inequalities and the extent to which they differ with time are important to explain the observed patterns at the national level, relevant for health monitoring. Of greater interest is the need to determine whether the prevalence of ANC among disadvantaged sub-groups (those aged less than 20, uneducated, in the poorest households, residing in rural areas, and in Alibori or Atacora) continues to worsen over time and whether that partly explains the decelerating trends.

Given its strong commitment to achieving equity in health, the WHO has developed several tools and resources to build and strengthen capacity for health inequality monitoring [24, 25]. Notable is a free and open-source software - Health Equity Assessment Toolkit (HEAT) - that facilitates the assessment and monitoring of within-country inequalities for several health outcomes, including ANC visits of at least four and with special emphasis on low-and-middle-income countries [25]. A set of simple and complex inequality measures can be computed with this software, and several documents and updates have been drafted for public use [25, 26]. This study applies HEAT by using selected simple and complex inequality measures to provide an in-depth understanding and perspectives on ANC patterns and their inherent disparities over time. Using data from three rounds of the Benin Demographic and Health Surveys (BDHS) (2006, 2011–12, 2017–18), the study seeks to answer two questions – (1) Are there inequalities in ANC coverage among women by age, place, region of residence, education, and economic status? (2) Have the levels of inequalities widened over time alongside the decline in Benin’s ANC coverage?

Our research investigates the trends of ANC coverage over time and its associated socio-demographic and economic inequalities in Benin. The findings are relevant to track the impact of existing programs and interventions on ANC and whether it is reaching the targeted audience. ANC is an essential element in the continuum of care for maternal and child health. Efforts could be strengthened to increase its uptake to ensure the attainment of the SDG targets for maternal, neonatal, infant and under-five mortality.

## Methods

### Study setting and data source

Data from the 2006, 2011–12, and 2017–18 BDHS was used for the study. The survey datasets are freely available to download via https://dhsprogram.com/data/dataset_admin/index.cfm. The BDHS is a nationwide survey conducted to ascertain periodic trends and changes in demographic indicators, health indicators, and social issues among men, women, and children [27]. A cross-sectional design was adopted for the BDHS, with the respondents sampled using a stratified multi-stage cluster sampling approach. The detailed sampling methodology has been highlighted in the BDHS report [27]. This study included women with a history of live birth five years before the survey. A sample of 10,522, 8,994, and 9,031 were extracted from the 2006, 2011–12, and 2017–18 BDHS, respectively, for inclusion in the study. The data from the 2006, 2011–12, and 2017–18 BDHS were available for use directly through the WHO HEAT online platform [26]. The Strengthening the Reporting of Observational Studies in Epidemiology (STROBE) checklist was considered when writing this paper [28].

### Variables

#### Outcome variable

The outcome variable of interest was four or more ANC visits. In the BDHS, childbearing women of reproductive age (aged 15–49) were asked about the number of visits made to the ANC clinic at a health facility for their recent pregnancy. A dichotomised variable with 0–3 ANC visits and four or more ANC visits was created and used in the analysis.

#### Dimension stratifiers

Five variables - age of the women, level of education, economic status (wealth quintile), type of place of residence, and sub-national region - were the inequality stratifiers used in the study, as found in existing literature [29]. These stratifiers were available in the WHO HEAT software for assessing inequalities in several health and social indicators [26]. The categories of each stratifier included women’s age (15–19 and 20–49), level of education (no education, primary, and secondary and above), economic status (poorest, poorer, middle, richer, and richest), place of residence (rural and urban), and sub-national region (Alibori, Atacora, Atlantique, Borgou, Collines, Couffo, Donga, Littoral, Mono, Oueme, Plateau, and Zou). For women’s age, the age groups were dichotomized to ascertain the extent to which inequalities in ANC visits are skewed against adolescents since they often face several forms of social and economic barriers compared to older women.

### Statistical analyses

We used the WHO HEAT online version [26] for all analyses. The WHO HEAT is an online statistical tool for analyzing health disparities within and between countries based on a variety of health and social indicators [26]. Detailed description of the WHO HEAT statistical package can be found in the literature [24–26]. Using four inequality measures, we examined the coverage of four or more ANC visits across the five inequality stratifiers: age, place of residence, economic status, level of education, and sub-national region. The four measures include difference (D), ratio (R), population attributable fraction (PAF), and population attributable risk (PAR). The formulas are shown below. D and PAR are absolute measures of inequality while R and PAF are relative measures. D and R double are simple measures, while PAR and PAF are complex measures.

$$D = AN{C_4}\,in \,most\,advantaged - AN{C_4}\,in \,most\,disadvantaged$$

$$R = \frac{{AN{C_4}\,in \,most\,advantaged}}{{AN{C_4}\,in \,most\,disadvantaged}}$$

$$PAR = AN{C_4}\,in \,most\,advantaged - \mu$$

$$PAF = \,\,\frac{{PAR}}{\mu }\,\,*\,\,100$$

Where $\mu$ is Benin’s national average estimate for ANC visits of four or more.

The inequalities between the extremes were prioritized for stratifiers with more than two categories. For instance, between no education and secondary or higher; between poorest and richest quintiles; and between Littoral and Atacora. The *most advantaged* in the formulas above correspond to age group 20–49, urban, secondary/higher education, richest, and Littoral, following evidence reported in existing literature on at least four ANC visits in Benin [13, 21, 23]. PAR and PAF were of utmost interest as they account for distribution across all subgroups (essential for stratifiers such as education, wealth, and sub-regions with more than two categories). The inequality metrics’ precise significance, calculation, and interpretation have been highlighted in the literature [24, 26]. All figures were generated using R programming.

### Ethical consideration

No ethical clearance was sought for this study because the BDHS dataset is freely available in the public domain. Permission to use the dataset for publication was obtained from the Monitoring and Evaluation to Assess and Use Results Demographic and Health Surveys (MEASURE DHS). The detailed ethical issues per the BDHS can be accessed via https://dhsprogram.com/Methodology/Protecting-the-Privacy-of-DHS-Survey-Respondents.cfm.

## Results

### Trends in antenatal care coverage of at least four visits

Table 1 shows the trends in ANC coverage by the various inequality dimensions considered in this study, spanning 2006 to 2017–18. In all, ANC coverage declined from 60.5% (in 2006) to 52.1% (in 2017–18). ANC coverage was relatively high among women aged 20–49 in 2006 (60.9%). This declined to 52.7% in 2017–18 but was still higher than the proportion for women aged 15–19 who had ANC in 2017–18 (47.3%). Women in richest households dominated in ANC coverage in all the survey waves (i.e. 87.5%, 76.6% and 75.5% in 2006, 2011 and 2017 respectively), whereas those in the lowest quintile had the least coverage in the same period (39.5%, 38.2%, and 31.6% in 2006, 2011–12 and 2017–18 respectively). Most women with at least secondary education had higher ANC coverage in 2006 (85.1%) and 2017–18 (70.2%). Conversely, those without formal education recorded the lowest prevalence in 2006 (53.7%) and 2017–18 (45%). Urban residents had more ANC visits throughout the period studied (i.e. 71.3%, 65.9% and 58.9% for 2006, 2011–12 and 2017–18, correspondingly). For region, the highest ANC visits occurred in Littoral region, both in 2006 (89.5%) and 2017–18 (81.9%).

**Table 1:** Trends in antenatal care coverage of at least four visits (2006–2017)

	2006 (60.5%)		2011–12 (58.2%)		2017–18 (52.1%)	
Dimension	*n*	%	*n*	%	*n*	%
**Age**						
15–19 years	1158	57.7 [53.9, 61.4]	908	57.4 [53.9, 60.9]	1087	47.3 [43.6, 51.1]
20–49 years	9364	60.9 [59.1, 62.6]	8086	58.3 [56.8, 59.7]	7944	52.7 [50.8, 54.6]
**Economic Status**						
Quintile 1 (poorest)	2214	39.5 [36.7, 42.5]	1813	38.2 [35.5, 41]	1827	31.6 [28.3, 35.1]
Quintile 2	2082	50.1 [47.3, 52.9]	1803	48.4 [45.6, 51.2]	1827	44.2 [41, 47.4]
Quintile 3	2174	58.4 [55.3, 61.3]	1786	58 [55.3, 60.6]	1837	51.0 [48, 54]
Quintile 4	2181	70.9 [68.6, 73.1]	1800	70.2 [67.6, 72.6]	1857	59.8 [56.7, 62.7]
Quintile 5 (richest)	1871	87.5 [85.8, 89]	1792	76.6 [73.5, 79.3]	1683	75.5 [72.6, 78.1]
**Education**						
No education	7690	53.7 [51.7, 55.7]	6328	51.3 [49.6, 52.9]	5807	45.0 [42.7, 47.2]
Primary school	1946	76.3 [74, 78.4]	1510	72.3 [69.8, 74.7]	1650	59.8 [56.9, 62.5]
Secondary school +	885	85.1 [82.2, 87.6]	1156	77.8 [74.8, 80.4]	1573	70.2 [67.4, 72.9]
**Place of residence**						
Rural	6779	54.6 [52.3, 56.9]	5318	52.9 [51.2, 54.6]	5508	47.7 [45.4, 50]
Urban	3742	71.3 [68.6, 73.9]	3675	65.9 [63.6, 68.2]	3523	58.9 [55.8, 61.9]
**Sub-region**						
Alibori	935	34.9 [26.6, 44.2]	601	34.5 [28.7, 40.8]	1226	45.1 [38.3, 52.2]
Atacora	751	35.1 [30.3, 40.2]	844	36.3 [31.8, 41.1]	800	26.1 [22, 30.5]
Atlantique	1166	73.7 [70, 77]	109	63.3 [60, 66.6]	1043	71.0 [67.2, 74.4]
Borgou	1033	51.9 [42.9, 60.7]	691	44.3 [39.8, 48.9]	1164	28.6 [23.3, 34.6]
Collines	761	62.2 [56.4, 67.7]	582	57.3 [51.9, 62.5]	604	49.8 [45.1, 54.4]
Couffo	863	52.0 [47.4, 56.4]	651	62.7 [58, 67.1]	583	53.1 [47, 59.1]
Donga	439	52.5 [43.7, 61.1]	386	45.4 [37.6, 53.3]	599	31.6 [25.9, 37.8]
Littoral	771	89.5 [86.5, 91.9]	1073	73.3 [68.7, 77.4]	408	81.9 [78, 85.1]
Mono	636	56.2 [50.5, 61.8]	489	68.5 [63.2, 73.4]	393	64.1 [58.4, 69.3]
Oueme	1438	72.3 [68.9, 75.5]	1141	75.4 [72.3, 78.3]	798	70.5 [65.7, 74.8]
Plateau	543	59.5 [53.3, 65.5]	622	42.4 [37.6, 47.3]	550	48.5 [42.8, 54.2]
Zou	1185	69.5 [65.2, 73.5]	81	68.2 [64.7, 71.6]	863	75.7 [71.6, 79.3]

### Inequalities in antenatal care coverage of at least four visits (2006–2017–18)

The following sections delve into the differences in ANC visits of at least four by key demographic and socioeconomic factors: age, economic status, education, place of residence and sub-national region. The sections below reveal inherent inequalities and the extent to which they have changed over the years.

### Age

**Fig. 1:**
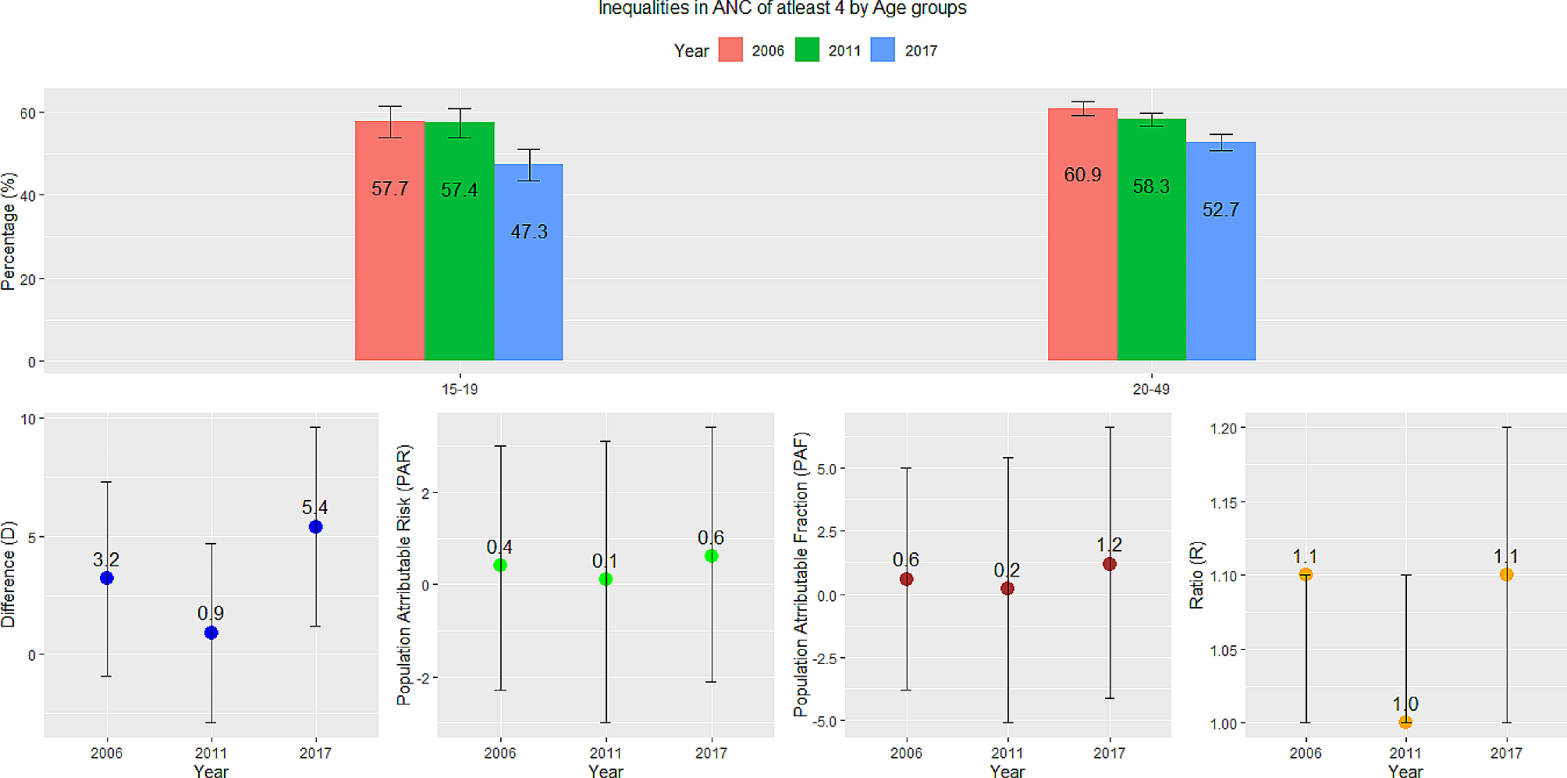
Trends and inequalities in ANC of at least four in Benin by age groups

Figure 1 shows the coverage of ANC visits of at least four and how the inequalities that exist between age groups have changed over time. In both age groups, there has been a decline in ANC visits and the inequality measures reveal that the disparities have not been substantial. The difference (D) between age groups was only 3.2% in 2006 and 5.4% in 2017–18. The ratio (R) was 1.1 in both 2006 and 2017. The PAF reveals the potential improvement in the national coverage of ANC of at least four that could be achieved if women aged 15–19 had the same level of coverage as those aged 20–49 years. The national average could have only been 0.6% higher in 2006 and 1.2% higher in 2017–18. The PAR also showed similar trends. Despite the decrease in ANC coverage in Benin, the evidence showed that age-related inequalities are very minimal.

### Economic status

**Fig. 2:**
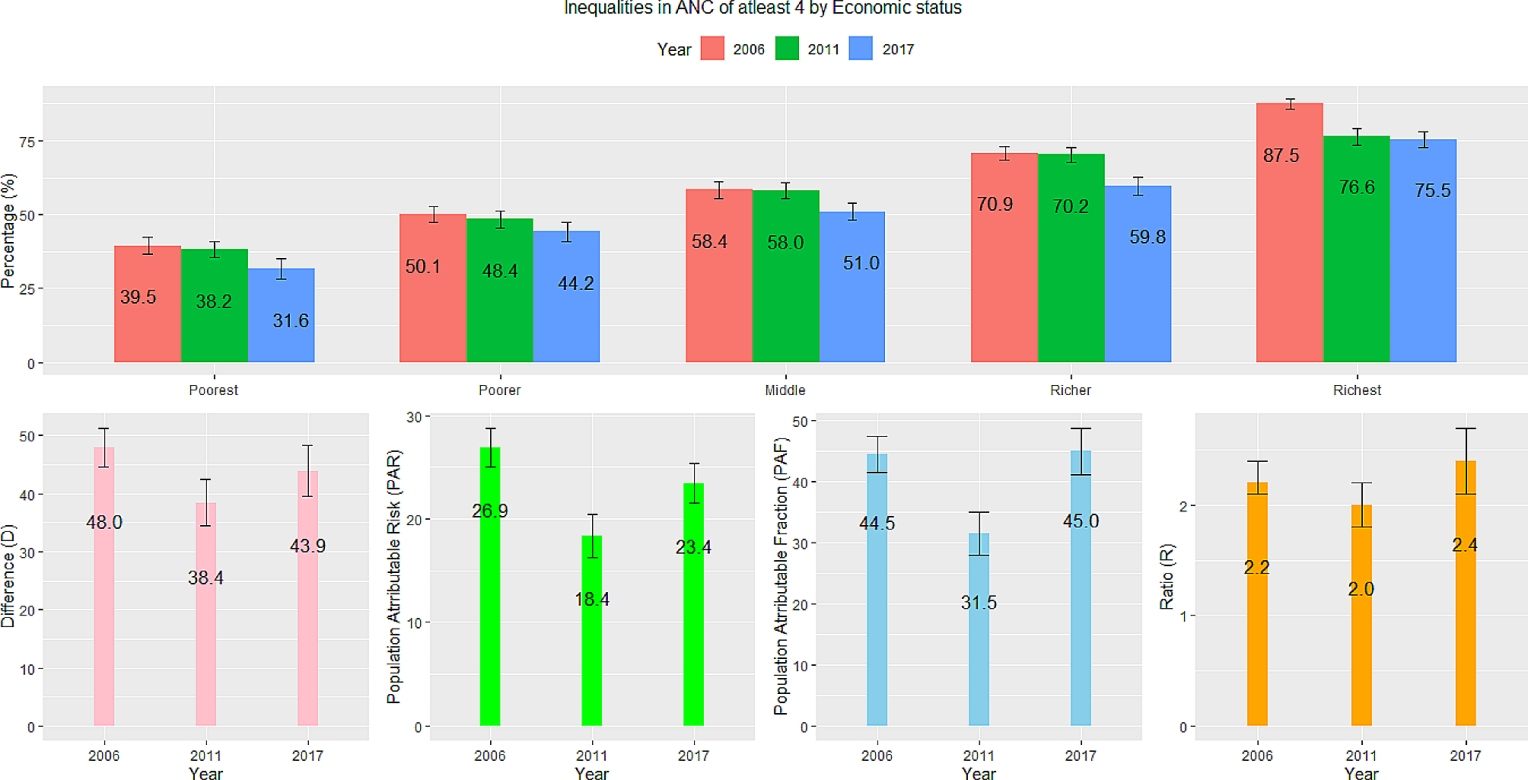
Trends and inequalities in ANC of at least four in Benin by economic status

Figure 2 shows results on ANC visits of at least four disaggregated by economic status for three years (2006, 2011–12, and 2017–18). Large disparities existed between women in the poorest and richest households. The difference in ANC visits between women in the richest and poorest households was almost 50% in 2006. This reduced to 38.4% in 2011–12 and increased to 43.9% in 2017. The PAR also followed similar trends, showing that Benin’s ANC coverage could have been 26.9% points higher in 2006, 18.4% points higher in 2011–12, and 23.4% points higher in 2017–18 if economic-related inequalities were eliminated. The PAF estimate for 2017–18 (45%) was not very different from the estimate in 2006 (44.5%). The ratio indicated that ANC attendance for women in the richest households was 2.2 times higher than those in the poorest households in 2006. It slightly increased to 2.4 times in 2017–18. The 95% confidence intervals for all four measures revealed that the patterns have been stable and highlight that economic-related inequalities have not improved over the period and could partly contribute to the decelerating national average trends.

### Education

**Fig. 3:**
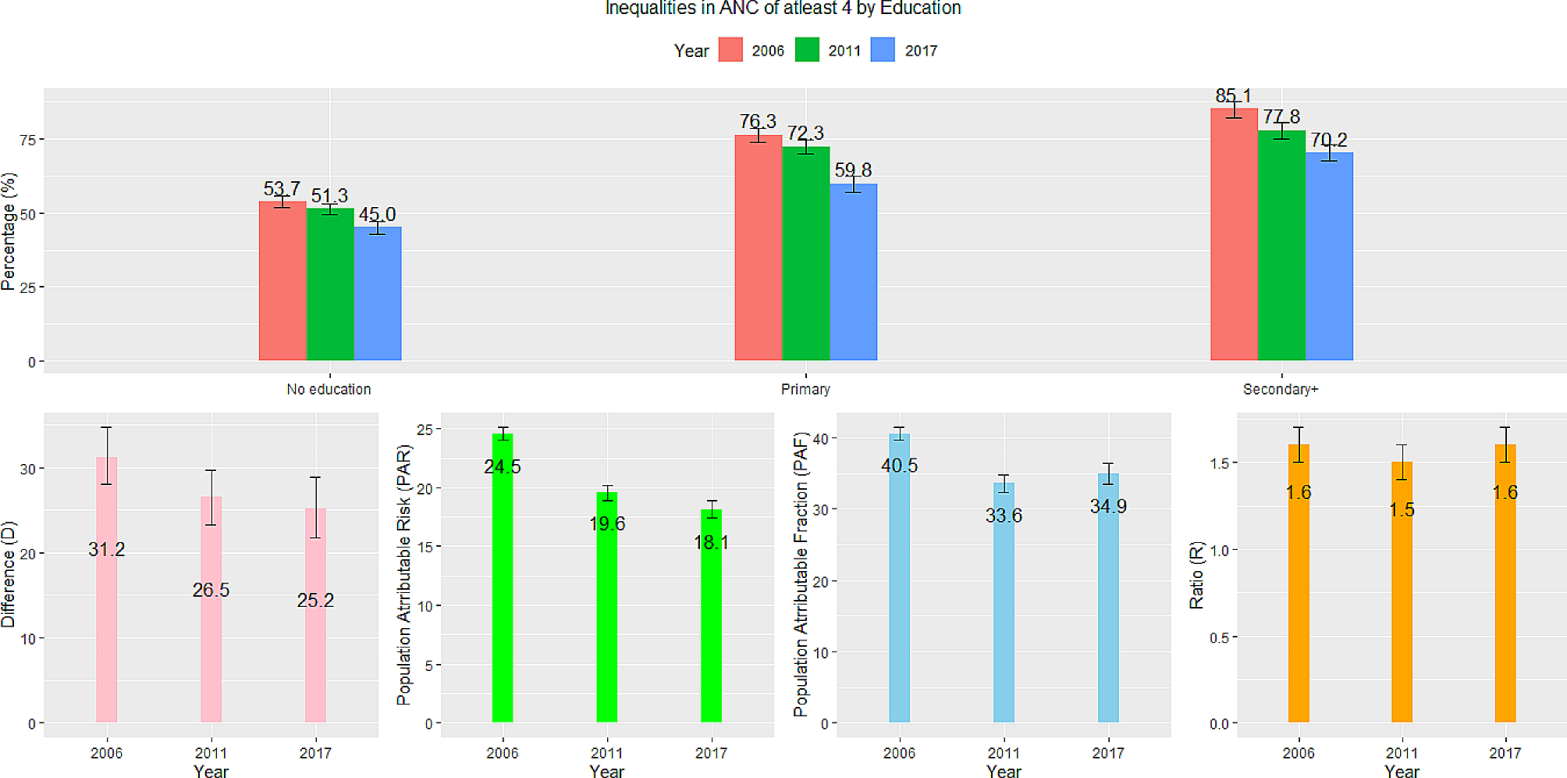
Trends and inequalities in ANC of at least four in Benin by education

Education-related inequalities in ANC visits are highlighted in Fig. 3. In 2006 and 2017–18, ANC visits of at least four was 1.6 times higher for women with secondary or higher education than those without. The difference in ANC visits between women with secondary education or higher and those without education was 31.2% in 2006 but decreased to 25.2% in 2017–18. The national average of ANC visits could have been 40.5% higher in 2006 and 35% higher in 2017 if there were no education related inequalities (PAF). The PAR followed a similar decreasing trend over time. While there has been a decline, the magnitude of education-related inequalities is still high, and efforts need to be strengthened to reduce them.

### Place of residence

**Fig. 4:**
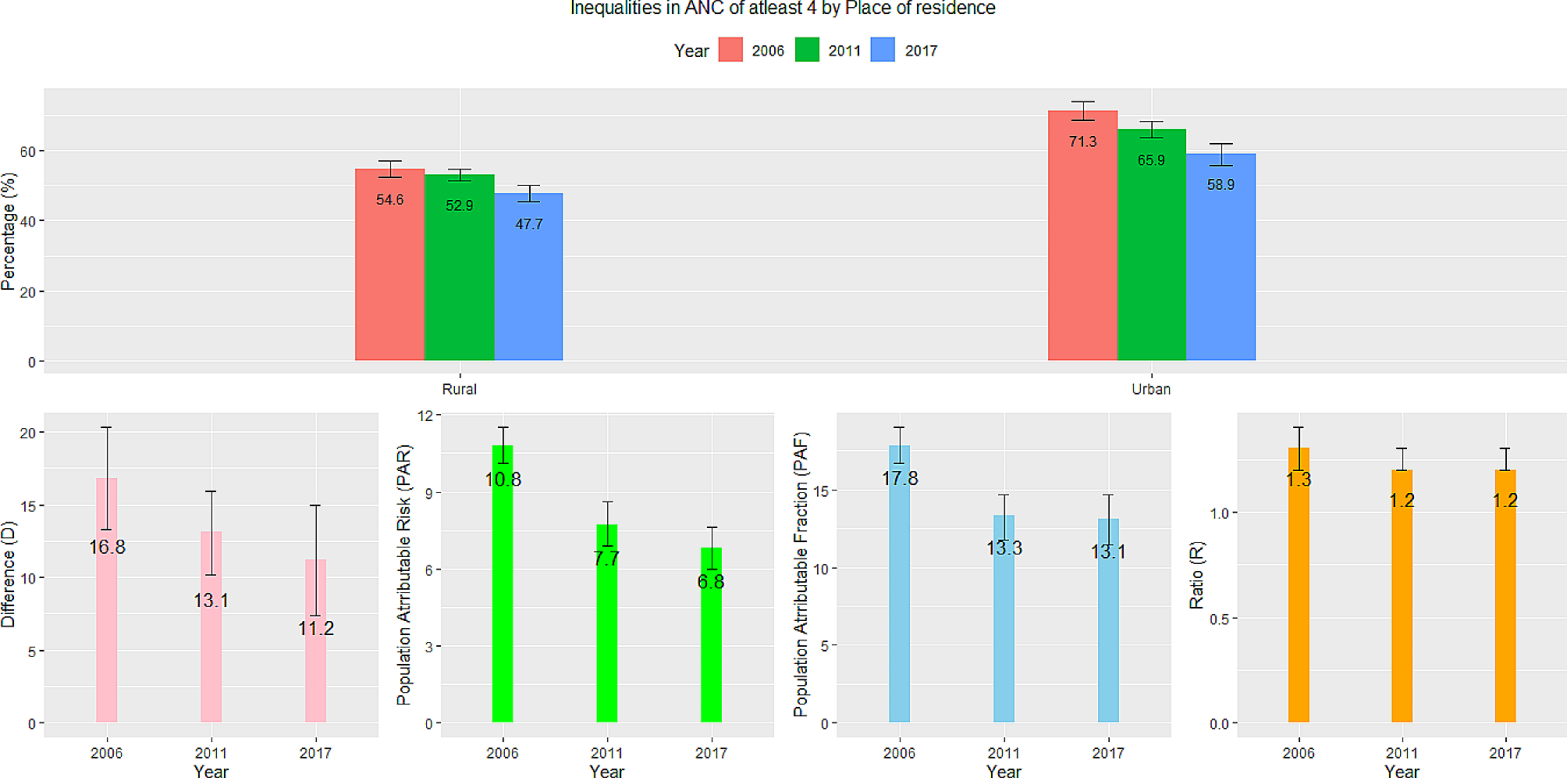
Trends and inequalities in ANC of at least four in Benin by place of residence

As illustrated in Fig. 4, inequalities in ANC visits disaggregated by women’s place of residence show substantial differences between rural and urban areas, despite a decline between 2006 and 2017–18. In 2006, an additional 13 per 100 women in urban areas had attended ANC on at least four counts compared to those in rural areas. This decreased to 11 per 100 in 2017. ANC among women in urban areas was 1.3 and 1.2 times higher compared to those in rural areas in 2006 and 2017–18, respectively. If the national average were to improve to a level where ANC coverage for rural women was at par with those in urban areas, the 2006 national estimate needed to be 24.5% points higher and for 2017–18, 18.1% points higher (PAR). The PAF also showed similar trends. The 95% confidence intervals emphasize that the PAR and PAF estimates for the 2006 and 2017–18 surveys are distinctively different, indicating a significant decline with time.

### Region of residence

**Fig. 5:**
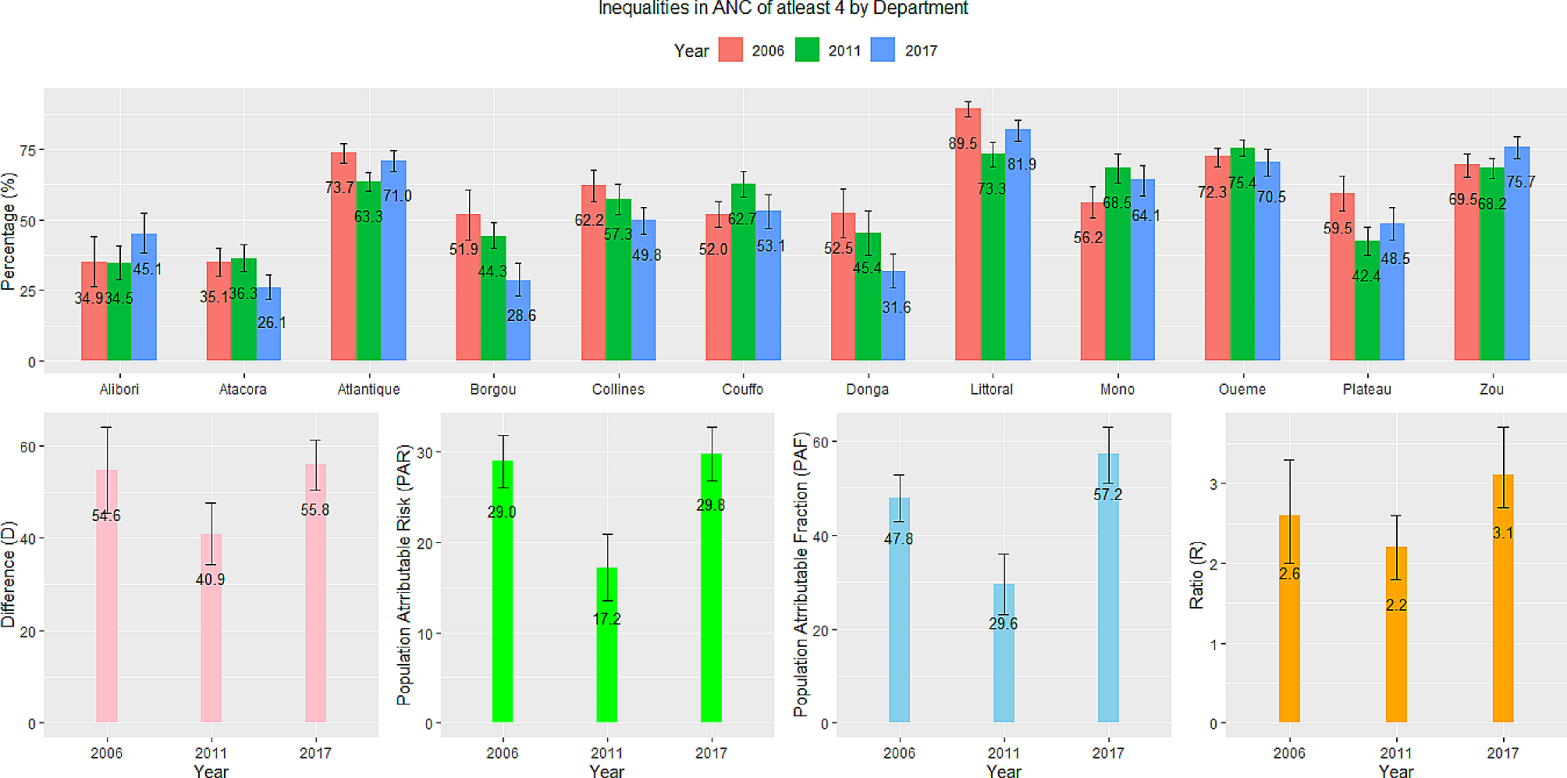
Trends and inequalities in ANC of at least four in Benin by region of residence

Region-related inequalities are shown in Fig. 5. Littoral had the highest coverage of ANC visits and Atacora, the lowest. Other regions had varying patterns. Alibori and Zou saw an increase in coverage from 34.9 to 69.5% in 2006 to 45.1 and 75.7% in 2017–18, respectively, while others such as Mono, Oueme, and Couffo saw an initial increase between 2006 and 2011–12 and a decrease afterward. The four measures revealed large region-related inequalities. The difference in the coverage of ANC between Littoral and Atacora was 54.6% in 2006 and 55.8% in 2017–18. The ratio of Litorral to Atacora increased from 2.6 in 2006 to 3.1 in 2017–18. The PAF and PAR had similar patterns, showing that region-based inequalities have increased over time. Without such inequalities, the national estimate could have been 47.8% higher in 2006 and 57.2% higher in 2017–18. Though the relative measures show an increase over time, the 95% confidence intervals reveal the differences across surveys could only be due to chance and perhaps the existing inequalities have stalled within a defined range.

## Discussion

In this study, we assessed the trends and inequalities in ANC coverage among women in Benin. The results show an 8.4% decline in ANC attendance (at least four) between 2006 and 2017–18. Unlike Benin’s performance, other sub-Saharan African countries such as Ghana [29] and Ethiopia [30] experienced substantial improvements. The decline highlights weakened maternal healthcare efforts, especially ANC interventions and programs. Given ANC’s relevance for monitoring maternal/ fetal health, providing vital health education, and identifying potential complications early in pregnancy [5], its decline connotes missed opportunities for timely intervention that could contribute to adverse maternal and neonatal health outcomes.

We found evidence for varying inequalities disaggregated by selected demographic and socioeconomic characteristics – age, economic status, education, place of residence, and region of residence. Sub-groups with low coverage of ANC visits in 2006 continued to experience the lowest uptake compared to their counterparts in 2017–18, in the face of the overall national decline. They included women aged 15–19, those with no education, in the poorest households, in rural areas, and in Atacora. Deviating from this decline was the Alibori region, which had the second lowest coverage in 2006 (34.9%) but saw a substantial 10% increase in 2017–18 (45.1%) and surpassing other regions such as Borgou and Donga. Inequality measures for the selected demographic and socioeconomic characteristics revealed two major patterns – an increase or a decrease. Region-related inequalities slightly increased over time, while education and rural-urban inequalities decreased. The patterns of economic-related inequalities were unclear, while age-related inequalities were negligible.

Region-related inequalities were the largest compared to the other socioeconomic and demographic factors, slightly increasing between 2006 and 2017–18. Though we report an overall decline in ANC, we found that Atacora had a slightly steeper decline between 2006 and 2017–18 (10%), compared to Littoral (7.6%) showing that women in the former region had been more affected. The magnitude of the inequalities is underpinned by several economic, social, and health factors that characterize the various regions. Littoral is majorly urban and houses Cotonou, the largest city in Benin. According to Benin‘s SDG monitoring report, Littoral ranks highest for access to public services [31] - accessibility and quality of health and education services, drinking water, electricity, internet, and civil registry. In fact, 99.2% of all childbirth deliveries were assisted by qualified health personnel and only 1.5% of its population were below the poverty line [31]. The region presents itself as a prime safety net for quality life and consequently reflects in its consistently high ANC coverage. On the other hand, Atacora (located in the Northern part of Benin) scored low on almost all public service indices while housing a substantial proportion of its population in extreme poverty [31], reflecting in the subsequent low coverage of ANC. Addressing the inequality gaps will, therefore, require public and health facility facelifts (structural and non-structural) in deprived regions such as Atacora. The facelifts could also be accompanied by additional research inquiries into socio-cultural and economic issues that reflect the social realities in various regions, and which underpin their respective ANC performance. The findings from such research can feed into implementing locally feasible and acceptable interventions to women in respective regions.

Our study revealed that women who belonged to the richest wealth quintile consistently had the highest ANC coverage compared to those in the poorest wealth quintile. The difference (D), ratio (R), PAR, and PAF also showed substantial economic-related inequalities in ANC coverage. The absolute measures (D and PAR) revealed a decline between 2006 and 2017–18 while the relative measures indicated a marginal increase, inferring well-grounded disparities. Our findings echo results from other studies that report favourable ANC outcomes for economically well-off women [10, 13, 20–22, 32] and further add that the disparities have not reduced over the period. Indirect maternal healthcare costs often serve as a disincentive for poorer women to seek ANC services [33] and constitute a major economic barrier. This underscores the need for the Benin government to enforce adequate measures to make maternal care not only more affordable but also easily accessible. Costs associated with traveling to health facilities constitute a higher proportion of indirect costs. They can be greatly reduced if health facilities are closer to women, especially those in deprived areas. Primary healthcare initiatives such as the Community-Based Health Planning and Services, which has been implemented in countries like Ghana [29], could be a great roadmap.

The trend analysis, consistent with previous studies [29, 30, 34], indicated that women with secondary or higher education had higher ANC coverage than those without or only primary education. Education empowers women by enhancing their knowledge about maternal health, reproductive rights, and the importance of seeking ANC services [35]. Educated women may also have better access to information through media and other channels, leading to increased awareness of the benefits of ANC, thereby informing their healthcare decision-making [30]. Conversely, women with no or only primary education may face barriers in accessing ANC services due to limited awareness, lower health literacy, and reduced decision-making power within their households. Although ANC coverage was consistently low among those with no formal education, the findings suggest an improvement in the inequality gap, as seen in the inequality estimates. The reduced gap is partly because the decline in ANC attendance between 2006 and 2017–18 was more rapid for women with secondary or higher education (~ 15%: 85.1% in 2006 and 70.2% in 2017–18) than those with no education (~ 9%: 53.7% in 2006 and 45.0% in 2017–18). More women with secondary or higher education are missing out on ANC visits, driving the inequality gap downwards. We recommend additional research to understand why this is the case and call for more concerted efforts to facilitate further narrowing of the inequality gap.

Across the three surveys, urban dwelling women had higher ANC coverage than their counterparts in rural areas. This finding aligns with evidence from prior studies conducted in Benin [13], Ethiopia [30] and Ghana [29, 32]. A possible explanation is that women in rural areas often face challenges related to inadequate and poorly equipped health facilities, often located far from their communities [13, 36]. These limited resources result in a shortage of skilled health attendants, leading to higher ANC dropouts. Another perspective is that rural dwelling women may exhibit a higher susceptibility to the impact of cultural norms and social beliefs, which in turn may discourage their engagement with skilled maternal care services including ANC [37]. The inequality estimates reveal a seemingly marginal decline in rural-urban inequalities between 2006 and 2017–18. Similar to what we observed for women’s education, we find that the difference in the proportion of urban women that attended ANC of four visits or more between 2006 and 2017–18 was higher (~ 12%: 71.3% in 2006 and 58.9% in 2017) than what was reported for rural dwelling women (~ 7%: 54.6% in 2006 and 47.7% in 2017–18). The resulting lower inequality gap over time could be partly driven by the fact that proportionally, more urban women are failing to meet the recommended four visits. Further research is needed to uncover the dynamics, while exploring its intersectionality with women’s education.

Even though adolescents have been considered a vulnerable group in maternal healthcare utilization due to limited autonomy and social stigmatization, the results from our analysis showed negligible inequalities in comparison to older women. Yaya et al.’s study corroborates our findings [13]. Though the odds of ANC visits were higher for women aged 20–49 compared to adolescents [15–19] in their study, the findings were insignificant [13]. In other contexts, the results were mixed [20, 22, 34] with associative effects ranging between weak and moderate. Our findings infer that Benin’s efforts to enhance adolescent-friendly services are paying off. At least for ANC, the gap isn’t extremely wide between those aged 20 years and above and adolescents.

Beyond the inequalities observed, our study highlights a decline in ANC coverage not only at the national level but also across subgroups. This could be an indication of national-level structural or financing barriers affecting maternal healthcare utilization in general. Efforts to address such barriers through policy design, implementation research, and health interventions need to be strengthened and sensitive to existing inequalities that make some women disadvantaged.

### Implications for policy and practice

Our findings underscore the urgent need for targeted policy interventions to reverse the decline in ANC coverage observed in Benin. Policymakers should prioritize maternal health and allocate sufficient resources to strengthen ANC interventions and programs. Specifically, investments should be directed towards improving access to ANC services in rural and economically disadvantaged regions such as Atacora. This may entail infrastructure upgrades, such as the construction of health facilities and the deployment of skilled health personnel to ensure equitable access to quality ANC services across all regions.

The substantial socioeconomic inequalities in ANC coverage revealed in our study necessitate interventions to reduce financial barriers to maternal healthcare access. To address the socioeconomic inequalities, the government of Benin should consider implementing policies to make maternal care more affordable and accessible, especially for women from low-income households. Measures such as subsidizing healthcare costs and expanding coverage of health insurance schemes could help alleviate the financial burden on disadvantaged women and improve their utilization of ANC services. Additionally, initiatives like the Community-Based Health Planning and Services, as successfully implemented in Ghana [29], could serve as a blueprint for community-based primary healthcare delivery in Benin, particularly in rural areas.

Efforts to promote girls’ education and literacy should be prioritized as part of broader strategies to improve maternal healthcare utilization. Investing in educational programs that enhance women’s knowledge about maternal health, reproductive rights, and the importance of ANC can empower women to make informed healthcare decisions and seek timely ANC services. Furthermore, targeted health education campaigns, utilizing various media channels, can raise awareness about the benefits of ANC among women with lower levels of education, thereby reducing disparities in ANC coverage.

### Strengths and limitations

Estimating inequalities is a cumbersome task; however, using the WHO HEAT software provides an appropriate medium for effective estimation. The DHS was based on a two-stage sampling methodology that ensures that the data is representative at regional and national levels. Hence, we extrapolate the findings to the wider population of women of reproductive age in Benin. Nevertheless, there were some limitations. Although the HEAT estimates inequalities, it cannot explain why inequalities exist [38]. This means that the reasons provided for the observed inequalities are based on prior literature and assumptions. Also, this study utilized data on available variables in the WHO HEAT software. Other factors such as cultural disparities that could influence ANC coverage were not accounted for. Hence, the inferences made from this study should be based on the available variables or inequality stratifiers. To mitigate this limitation, future research could incorporate qualitative methodologies such as interviews or focus groups to explore the socio-economic, cultural, and systemic factors contributing to the observed inequalities. We acknowledge that our study’s descriptive nature precludes the establishment of causal relationships. Moving forward, longitudinal studies or randomized controlled trials could be employed to elucidate causal pathways and better understand the mechanisms driving disparities in ANC coverage among women in Benin.

## Conclusion

ANC coverage in Benin has declined over the years by 8.4%, with pervasive inequalities by wealth index, educational levels, rural-urban, and regional residence that are favorable to those in the richest wealth quintile, urban dwelling women, and those with secondary or higher education. A one-size-fit approach to narrow inequalities related to ANC coverage would not be feasible in Benin. Rather, the Benin government could devise comprehensive strategies encompassing both supply and demand-side interventions, focusing on reaching the uneducated population residing in rural areas and Atacora as well as those in the poorest households.

## Data Availability

No datasets were generated or analysed during the current study.

## Abbreviations

ANC: Antenatal Care
BDHS: Benin Demographic and Health Survey
D: Difference
HEAT: Health Equity Assessment Toolkit
PAF: Population Attributable Fraction
PAR: Population Attributable Risk
R: Ratio
SDG: Sustainable Development Goal
STROBE: Strengthening the Reporting of Observational Studies in Epidemiology
WHO: World Health Organization

## References

[1] Kuhnt J, Vollmer S. Antenatal care services and its implications for vital and health outcomes of children: evidence from 193 surveys in 69 low-income and middle-income countries. BMJ Open. 2017;7(11):e017122. 29146636 10.1136/bmjopen-2017-017122 PMC5695442

[2] Tekelab T, Chojenta C, Smith R, Loxton D. The impact of antenatal care on neonatal mortality in sub-saharan Africa: a systematic review and meta-analysis. PLoS One. 2019;14(9):e0222566. 31518365 10.1371/journal.pone.0222566 PMC6743758

[3] Turi E, Fekadu G, Taye B, Kejela G, Desalegn M, Mosisa G, et al. The impact of antenatal care on maternal near-miss events in Ethiopia: a systematic review and meta-analysis. Int J Afr Nurs Sci. 2020;13:100246.

[4] Wondemagegn AT, Alebel A, Tesema C, Abie W. The effect of antenatal care follow-up on neonatal health outcomes: a systematic review and meta-analysis. Public Health Rev. 2018;39(1):33. 30574407 10.1186/s40985-018-0110-y PMC6296103

[5] World Health Organization. WHO recommendations on antenatal care for a positive pregnancy experience [Internet]. Geneva: World Health Organization. 2016 [cited 2023 May 2]. 152 p. https://apps.who.int/iris/handle/10665/250796. 28079998

[6] Cherie N, Abdulkerim M, Abegaz Z, Walle Baze G. Maternity continuum of care and its determinants among mothers who gave birth in Legambo district, South Wollo, northeast Ethiopia. Health Sci Rep. 2021;4(4):e409. 34754945 10.1002/hsr2.409 PMC8562404

[7] Kerber KJ, de Graft-Johnson JE, Bhutta ZA, Okong P, Starrs A, Lawn JE. Continuum of care for maternal, newborn, and child health: from slogan to service delivery. Lancet. 2007;370(9595):1358–69. 17933651 10.1016/S0140-6736(07)61578-5

[8] Seidu AA, Ahinkorah BO, Aboagye RG, Okyere J, Budu E, Yaya S. Continuum of care for maternal, newborn, and child health in 17 sub-saharan African countries. BMC Health Serv Res. 2022;22(1):1394. 36419060 10.1186/s12913-022-08693-w PMC9682703

[9] United Nations Children’s Fund. UNICEF DATA. 2022 [cited 2023 May 2]. Antenatal care. https://data.unicef.org/topic/maternal-health/antenatal-care/.

[10] Adedokun ST, Yaya S. Correlates of antenatal care utilization among women of reproductive age in sub-saharan Africa: evidence from multinomial analysis of demographic and health surveys (2010–2018) from 31 countries. Arch Public Health. 2020;78(1):134. 33317637 10.1186/s13690-020-00516-w PMC7737303

[11] Tessema ZT, Tesema GA, Yazachew L. Individual-level and community-level factors associated with eight or more antenatal care contacts in sub-saharan Africa: evidence from 36 sub-saharan African countries. BMJ Open. 2022;12(3):e049379. 35273040 10.1136/bmjopen-2021-049379 PMC8915341

[12] Gryseels C, Dossou JP, Vigan A, Boyi Hounsou C, Kanhonou L, Benova L, et al. Where and why do we lose women from the continuum of care in maternal health? A mixed-methods study in Southern Benin. Trop Med Int Health. 2022;27(3):236–43. 35098607 10.1111/tmi.13729 PMC9306704

[13] Yaya S, Uthman OA, Amouzou A, Ekholuenetale M, Bishwajit G. Inequalities in maternal health care utilization in Benin: a population based cross-sectional study. BMC Pregnancy Childbirth. 2018;18(1):194. 29855277 10.1186/s12884-018-1846-6 PMC5984297

[14] Ekholuenetale M, Nzoputam CI, Barrow A, Onikan A. Women’s enlightenment and early antenatal care initiation are determining factors for the use of eight or more antenatal visits in Benin: further analysis of the demographic and Health Survey. J Egypt Public Health Assoc. 2020;95(1):13. 32813174 10.1186/s42506-020-00041-2 PMC7364685

[15] Alvarez JL, Gil R, Hernández V, Gil A. Factors associated with maternal mortality in Sub-saharan Africa: an ecological study. BMC Public Health. 2009;9(1):462. 20003411 10.1186/1471-2458-9-462 PMC2801510

[16] Tesema GA, Teshale AB, Tessema ZT. Incidence and predictors of under-five mortality in East Africa using multilevel Weibull regression modeling. Arch Public Health. 2021;79(1):196. 34772469 10.1186/s13690-021-00727-9 PMC8588577

[17] Konnon R, Semyatov S, Soyunov M, Sokhova Z, Zulumyan T. Trends on maternal mortality in the Republic of Benin and comparison with the neighboring countries. Med Law Soc. 2020;13(2):197–216. 10.18690/mls.13.2.197-216.2020

[18] United Nations Children’s Fund. UNICEF DATA. 2023 [cited 2023 Oct 30]. Benin - Demographics, Health & Infant Mortality. https://data.unicef.org/country/ben/.

[19] McCartney G, Popham F, McMaster R, Cumbers A. Defining health and health inequalities. Public Health. 2019;172:22–30. 31154234 10.1016/j.puhe.2019.03.023 PMC6558275

[20] Andegiorgish AK, Elhoumed M, Qi Q, Zhu Z, Zeng L. Determinants of antenatal care use in nine sub-saharan African countries: a statistical analysis of cross-sectional data from demographic and health surveys. BMJ Open. 2022;12(2):e051675. 35149562 10.1136/bmjopen-2021-051675 PMC8845176

[21] Dansou J, Adekunle AO, Arowojolu AO. Factors Associated with Antenatal Care services utilisation patterns amongst Reproductive Age women in Benin Republic: an analysis of 2011/2012 Benin Republic’s demographic and Health Survey Data. Niger Postgrad Med J. 2017;24(2):67. 28762359 10.4103/npmj.npmj_16_17

[22] Mamuye Azanaw M, Gebremariam AD, Teshome Dagnaw F, Yisak H, Atikilt G, Minuye B, et al. Factors Associated with numbers of Antenatal Care visits in Rural Ethiopia. J Multidiscip Healthc. 2021;14:1403–11. 34140778 10.2147/JMDH.S308802 PMC8203265

[23] United Nations Children’s Fund. Maternal and newborn health disparities - Benin. New York: UNICEF; 2018.

[24] World Health Organization. Handbook on health inequality monitoring: with a special focus on low-and-middle income countries. Geneva: World Health Organization; 2013.

[25] World Health Organization. Health Equity Assessment Toolkit Plus (HEAT plus): Software for exploring and comparing health inequalities in countries. Upload database edition. Geneva: World Health Organization; 2021.

[26] Hosseinpoor AR, Nambiar D, Schlotheuber A, Reidpath D, Ross Z. Health Equity Assessment Toolkit (HEAT): software for exploring and comparing health inequalities in countries. BMC Med Res Methodol. 2016;16(1):1–10. 27760520 10.1186/s12874-016-0229-9 PMC5069829

[27] INSAE ICF, Enquête Démographique. et de Santé au Bénin, 2017–2018 [Internet]. Cotonou, Bénin; Rockville, Maryland, USA: Institut National de la Statistique et de l’Analyse Économique (INSAE) et ICF; 2019 [cited 2023 Oct 30]. https://dhsprogram.com/pubs/pdf/FR350/FR350.pdf.

[28] Von Elm E, Altman DG, Egger M, Pocock SJ, Gøtzsche PC, Vandenbroucke JP. The strengthening the reporting of Observational studies in Epidemiology (STROBE) Statement: guidelines for reporting observational studies. Int J Surg. 2014;12(12):1495–9. 25046131 10.1016/j.ijsu.2014.07.013

[29] Seidu AA, Okyere J, Budu E, Duah HO, Ahinkorah BO. Inequalities in antenatal care in Ghana, 1998–2014. BMC Pregnancy Childbirth. 2022a;22(1):478. 35698085 10.1186/s12884-022-04803-y PMC9190076

[30] Tsegaye S, Yibeltal K, Zelealem H, Worku W, Demissie M, Worku A, et al. The unfinished agenda and inequality gaps in antenatal care coverage in Ethiopia. BMC Pregnancy Childbirth. 2022;22(1):82. 35093008 10.1186/s12884-021-04326-y PMC8801127

[31] UN Sustainable Development Solutions Network. Benin Sustainable Development Report 2022 [Internet]. Paris: UN Sustainable Development Solutions Network; 2022 [cited 2023 Oct 16]. https://s3.amazonaws.com/sustainabledevelopment.report/2022/2022-benin-sustainable-development-report-english.pdf.

[32] Asamoah BO, Agardh A, Pettersson KO, Östergren PO. Magnitude and trends of inequalities in antenatal care and delivery under skilled care among different socio-demographic groups in Ghana from 1988–2008. BMC Pregnancy Childbirth. 2014;14(1):295. 25169877 10.1186/1471-2393-14-295 PMC4155087

[33] Shibre G, Zegeye B, Idriss-Wheeler D, Ahinkorah BO, Oladimeji O, Yaya S. Socioeconomic and geographic variations in antenatal care coverage in Angola: further analysis of the 2015 demographic and health survey. BMC Public Health. 2020;20(1):1243. 32799833 10.1186/s12889-020-09320-1 PMC7429730

[34] Sakeah E, Okawa S, Rexford Oduro A, Shibanuma A, Ansah E, Kikuchi K, et al. Determinants of attending antenatal care at least four times in rural Ghana: analysis of a cross-sectional survey. Glob Health Action. 2017;10(1):1291879. 28578634 10.1080/16549716.2017.1291879 PMC5496066

[35] Ahuru RR. The influence of women empowerment on maternal and childcare use in Nigeria. Int J Healthc Manag. 2021;14(3):690–9. 10.1080/20479700.2019.1688505

[36] Bobo FT, Yesuf EA, Woldie M. Inequities in utilization of reproductive and maternal health services in Ethiopia. Int J Equity Health. 2017;16(1):105. 28629358 10.1186/s12939-017-0602-2 PMC5477250

[37] Mekonnen T, Dune T, Perz J. Maternal health service utilisation of adolescent women in sub-saharan Africa: a systematic scoping review. BMC Pregnancy Childbirth. 2019;19(1):366. 31638927 10.1186/s12884-019-2501-6 PMC6805384

[38] Kirkby K, Schlotheuber A, Vidal Fuertes C, Ross Z, Hosseinpoor AR. Health Equity Assessment Toolkit (HEAT and HEAT plus): exploring inequalities in the COVID-19 pandemic era. Int J Equity Health. 2022;21(3):172. 36471346 10.1186/s12939-022-01765-7 PMC9720922

